# Mitochondrial Dysfunction and **α**-Synuclein Synaptic Pathology in Parkinson's Disease: Who's on First?

**DOI:** 10.1155/2015/108029

**Published:** 2015-03-31

**Authors:** Michela Zaltieri, Francesca Longhena, Marina Pizzi, Cristina Missale, PierFranco Spano, Arianna Bellucci

**Affiliations:** ^1^Department of Molecular and Translational Medicine and National Institute of Neuroscience, University of Brescia, Viale Europa 11, 25123 Brescia, Italy; ^2^IRCCS San Camillo, Via Alberoni 70, 30126 Venice, Italy

## Abstract

Parkinson's disease (PD) is the most common neurodegenerative movement disorder. Its characteristic neuropathological features encompass the loss of dopaminergic neurons of the nigrostriatal system and the presence of Lewy bodies and Lewy neurites. These are intraneuronal and intraneuritic proteinaceous insoluble aggregates whose main constituent is the synaptic protein *α*-synuclein. Compelling lines of evidence indicate that mitochondrial dysfunction and *α*-synuclein synaptic deposition may play a primary role in the onset of this disorder. However, it is not yet clear which of these events may come first in the sequel of processes leading to neurodegeneration. Here, we reviewed data supporting either that *α*-synuclein synaptic deposition precedes and indirectly triggers mitochondrial damage or that mitochondrial deficits lead to neuronal dysfunction and *α*-synuclein synaptic accumulation. The present overview shows that it is still difficult to establish the exact temporal sequence and contribution of these events to PD.

## 1. Introduction

Parkinson's disease (PD) is a complex motor disorder whose clinical manifestations include a series of typical symptoms encompassing resting tremor, bradykinesia, akinesia, rigidity, and postural instability. It is ascertained that these clinical features are linked with the loss of dopaminergic neurons in the pars compacta of the substantia nigra. Lewy bodies (LBs), eosinophilic proteinaceous round-shaped inclusions and Lewy neurites (LNs), enlarged aberrant thread-containing neuritic structures, both of which are mainly composed by *α*-synuclein, are key hallmarks of PD and can affect the substantia nigra pars compacta and other brain areas. Although the majority of PD subjects account for a sporadic onset, about 10% of the cases present a familial inheritance [1]. At present, 18 different PARK loci have been associated to the onset of genetic forms of either autosomal dominant or autosomal recessive PD, but only a few of them, including *α*-synuclein, have been recognized as pathogenetic [2]. In recent years, there has been a tremendous growth of knowledge of the molecular basis of the neurodegenerative events participating to PD. Among them, apoptotic and nonapoptotic programmed cell death (PCD) [3], aberrant autophagic regulation [4], endoplasmic reticulum (ER) dysfunction [5], and intracellular calcium rise [6]. Nonetheless, their exact contribution to neuronal degeneration is still a matter of investigation. Interestingly, all these phenomena have been reported to constitute a link between *α*-synuclein deposition and the onset of impaired mitochondrial homeostasis, a process that is widely recognized to be central in PD pathogenesis [7]. This suggests that both *α*-synuclein pathology and mitochondrial impairment may very well constitute a dangerous duet whose critical interplay may affect neuronal resilience by participating with the above cited processes to neurodegeneration in PD. For instance, *α*-synuclein overexpression in neurons induces caspase-dependent nonapoptotic cell death associated with cytochrome c release from the mitochondria [8] while aberrant *α*-synuclein results in autophagy activation and in a reduction of the number and length of mitochondria that suggest the induction of autophagic mitochondrial removal (mitophagy) [4]. Accumulation of *α*-synuclein within the ER triggers the ER stress related pathway of the unfolded protein response (UPR) that coincides with cytochrome c release from the mitochondria [9] and finally calcium entry-related *α*-synuclein inclusions elevate dendritic mitochondrial oxidant stress in dopaminergic neurons [10]. Hence, *α*-synuclein pathology and mitochondrial damage seem to act as pivotal players in the pathogenesis of PD.

The relevance of *α*-synuclein deposition in the pathogenesis of PD is sustained by the fact that the staging of clinical progression of the disease has been correlated with the spreading of *α*-synuclein pathology in the brain [11–13]. In the early phases, LBs affect the dorsal motor nuclei and the reticular zone (Stage 1); then the pathology progresses to the medulla oblongata, pontine tegmentum, raphe nuclei, locus coeruleus, and reticular nucleus (Stage 2) and subsequently reaches the substantia nigra (Stage 3). This stage is thought to coincide with the onset of motor signs. In advanced phases, *α*-synuclein deposits can be found in basal mesencephalon, allocortex, neocortex, and the cortical transentorhinal region and premotor areas (Stage 5) and then proceed forward to first order as well as primary sensory association areas of the neocortex and premotor zone (stage 6). A link between the onset of clinical symptoms and *α*-synuclein pathology is further supported by studies indicating that a plethora of typical PD premotor signs encompassing olfactory dysfunctions, sleep disorders, cardiovascular dysfunction, and gastrointestinal and urinary abnormalities may still be attributed to *α*-synuclein accumulation affecting both central and peripheral neuronal cells [14].

It has been found that 90% or even more of *α*-synuclein aggregates in the brain of PD patients are located at the presynapses in the form of very small deposits. In parallel, dendritic spines are retracted, whereas the presynapses are relatively preserved, suggesting that neurotransmitter deprivation is an early event in PD pathogenesis. Several lines of evidence from rodent models of PD have confirmed that *α*-synuclein aggregation may damage neurons starting from the synaptic compartment and this leads to a consequent retrograde axonal damage terminating in neuronal cell degeneration with a dying back fashion [15, 16]. Therefore, the synaptic damage related to *α*-synuclein deposition could play a primary role in coaxing the onset of motor symptoms and neuronal loss in PD.

In this scenario, it is though relevant to consider that the polarity and correct functionality of neurons depend on an efficient transport of mitochondria to areas of high energy consumption such as presynaptic sites [17]. Furthermore, the selective vulnerability of substantia nigra pars compacta neurons in PD has been postulated to depend on the peculiar “energy-demanding” physiology of these cells [18]. Dopaminergic nigrostriatal neurons display an extended axonal field and the number of synapses for each axon is orders of magnitude higher than that of other neurons [19]. They generate autonomous action potentials by unusual engaging of L-type Ca^2+^ channels, thus requiring the activation of ATP-dependent Ca^2+^ pumps to maintain proper cytoplasmic Ca^2+^ homeostasis [20]. To afford this gargantuan energy demand, mitochondria and endoplasmic reticulum in substantia nigra pars compacta dopaminergic neurons generate reactive oxygen species (ROS) which are constantly neutralized by antioxidant systems [21]. However, nigral dopaminergic neurons, inter alia, are especially vulnerable to oxidative processes, as they present elevated amounts of iron but reduced antioxidative players [22, 23]. It is thus likely that factors disturbing the equilibrium between the energy demand and the consequent production of oxidative stress mediators may significantly impinge on the survival of these cells. In support of this hypothesis, it has been found that mice deficient in the PD-associated gene Parkin show neuronal as well as astrocytic mitochondrial alterations [24, 25]. Of note, respiratory dysfunction in astrocytes may lead to a reduction of the neurotrophic support during neuronal development, a phenomenon that could contribute to the induction of neuronal deficits [26]. Another protein whose mutations have been found to induce rare forms of autosomal recessive parkinsonism is DJ-1. This latter seems to function as a redox-sensitive molecular chaperone, whose loss of function may induce oxidative stress and consequently mitochondrial damage [27] that are associated with deficits in mechanisms to counteract reactive oxygen species (ROS) formation [28]. DJ-1 knockout animals show an enhanced sensitivity to the exposure of mitochondrial toxins; however, they do not develop PD-like pathological alterations* per se*. Instead, expression of mutant forms of leucine-rich kinase 2 (LRRK2), that are the most common cause for the onset of familial PD, only produces subtle alterations in mitochondria morphology and integrity* in vivo*, although it has been hypothesized that the protein may also regulate mitochondrial dynamics [27].

All these studies point toward mitochondrial dysfunction as a relevant pathological mechanism in the induction of neuronal dysfunction and degeneration in the PD brain. Normal mitochondrial activity is fundamental to maintain neuronal homeostasis and organelle turnover and defects in these processes can induce neurotoxicity through oxidative mechanisms or by promoting *α*-synuclein misfolding and oligomerization [29]. Therefore, whether *α*-synuclein synaptic deposition is the priming event for the onset of PD or vice versa mitochondrial deficits anticipate and trigger *α*-synuclein pathology and dopamine neuron degeneration is still an open question.

In this review we will describe the critical interplay between *α*-synuclein synaptic accumulation and mitochondrial dysfunction in PD and try to unfold which comes first in the pathogenesis of PD.

## 2. The Synaptic Pathology of ***α***-Synuclein in PD

In the PD brain, LBs are mainly found in sites of neuronal loss, that is, the substantia nigra and locus coeruleus, so they were hypothesized to play a pathogenic role in neuronal degeneration. However, despite the negative correlation occurring between nigral neuronal density and *α*-synuclein burden in the PD brain, this was found to be unrelated to no relationship with Hoehn and Yahr stage or disease duration [30]. These findings, although supporting that the severity of neuron degeneration in the substantia nigra is closely coupled to *α*-synuclein burden, have undermined the above presented hypothesis. Moreover, it has to be emphasized that the majority of degenerating neurons do not contain LBs. Neurons may show morphological, dendritic, and synaptic alterations or biochemical changes in the absence or presence of LBs. It may thus be feasible that accumulation of *α*-synuclein at synaptic sites rather than LBs formation may be the primary cause of neuronal death in PD. In line with this idea, a recent research report has highlighted that synaptic *α*-synuclein aggregates of orders of magnitude smaller than LBs are very abundant in the PD brain [31]. The amount of these small *α*-synuclein aggregates markedly exceeds that of LBs or LNs. Indeed, the LBs fraction contains 0.02–11% of their total amount whereas the 50–92% of them were found in synaptosomal protein extracts.

These lines of evidence hint that synaptic *α*-synuclein pathology could initiate and determine the onset of motor symptoms in PD. Indeed, clinical manifestations of the disease appear when dopamine levels in the striatum are reduced to 80% of normal levels, as measured by a decrease in [^18^F] fluoro-DOPA PET binding, a consequence of dopamine neuron loss in substantia nigra [15, 32, 33]. Of note, this initial symptomatic phase is characterized by a significant worsening of putaminal presynaptic deficiency, with a marked reduction in dopamine presynaptic storage, transporter binding, and release [34, 35]. These findings point toward the occurrence of a specific sequence of events, induced by *α*-synuclein accumulation and aggregation at synaptic sites, that could trigger synaptic dysfunction. Numerous studies have emphasized that *α*-synuclein finely tunes synaptic vesicle homeostasis and dopamine reuptake. For instance, the protein acts as a negative modulator of synaptic dopamine release and its overexpression and aggregation start from the synapse and induce striatal synaptic deficits, axonal damage, and motor impairment [36–40]. Alpha-synuclein aggregation can also affect the subcellular distribution of the dopamine transporter by reducing its membrane translocation [41–43]. This is in line with studies demonstrating that the striatal decrease of dopamine transporter labeling in the PD brain inversely correlates with nigral *α*-synuclein burden rather than with the presence of LBs [44]. In addition, specific changes in the distribution of synaptic proteins, such as soluble N-ethylmaleimide sensitive fusion attachment protein receptor (SNARE) proteins, have been observed in the postmortem brain of patients with early onset PD [45]. The fact that synaptic abnormalities may be generated by *α*-synuclein deposition is thus not surprising when considering that the protein functions as a key modulator in the control of dopamine synapse homeostasis [36, 46, 47]. In addition, transgenic overexpression of *α*-synuclein can rescue mice deficient in the synaptic cochaperone cysteine-string protein-*α* (CSP*α*) from neurodegeneration, indicating that the protein cooperates with CSP*α* to protect neuronal cells against injury at synaptic terminals [48].

Several studies have demonstrated that *α*-synuclein overexpression or aggregation at synaptic sites results in neuronal dysfunction by obstructing normal cellular trafficking and/or trapping cellular components in inappropriate locations [15, 49–52]. Of note, these synaptic, axonal, and dendritic alterations precede the onset of motor dysfunctions in numerous* in vivo* transgenic and viral-vector-based *α*-synuclein overexpressing models of PD [37, 39, 53, 54]. Therefore *α*-synuclein, which is enriched at synaptic sites, acts as a crucial modulator of synaptic vesicle related processes. It is thus possible to imagine that mutations, modifications, conformational changes, accumulation, and pathological deposition of *α*-synuclein in insoluble aggregates can first disrupt synaptic homeostasis and function. This event may then affect both axonal and dendritic trafficking, blocking protein and energy demand to synapses and inducing a critical accumulation of proteins within the cell body, resulting in proteasomal collapse, endoplasmic reticulum stress, intracellular calcium rise, and impaired mitochondrial efficiency. These findings suggest that it is more plausible to conclude that *α*-synuclein related synaptic pathology precedes the onset of mitochondrial dysfunction.

## 3. Mitochondrial Dysfunction in PD

A large body of evidence supports a central role of mitochondria alterations in the pathogenesis of PD. Epidemiological studies have shown that pesticides inhibiting mitochondrial Complex I and increasing oxidative stress are associated with PD [55, 56]. In addition, it is known since decades that 1-methyl-4-phenyl-1,2,3,6-tetrahydropyridine (MPTP), a neurotoxin widely used to generate PD models, inhibits NADH/ubiquinone oxidoreductase in the mitochondria electron transport system (ETS) [57].

Studies in rodent and primate models have demonstrated that dopamine neuron degeneration, central and peripheral *α*-synuclein pathology, and motor deficits can be induced by the systemic and local administration of this agent [57–60]. Likewise, rotenone and annonacin, which are mitochondria complex I inhibitors, or other pesticides acting on the mitochondrial ETS (paraquat, maneb, dieldrin, heptachlor, and atrazine) induce pathological, biochemical, and behavioral features of PD [61–67]. These lines of evidence point out that alterations of mitochondrial pathways are involved in the pathogenesis of parkinsonian-like syndromes. Of note, mitochondrial dysfunctions have been reported in muscle tissues, platelets, lymphocytes, and fibroblasts of PD subjects, thus supporting the idea that this phenomenon does not exclusively involve central neuronal cells and represents an important feature of PD [68–76]. In addition, mutations in PINK-1, a mitochondrial kinase involved in mitochondrial fission, mitophagy, and quality control [77–80], can lead to the onset of familial forms of PD [81, 82]. Postmortem findings have confirmed a reduction in the activity in complexes I and II of the ETS in the cortex and substantia nigra of sporadic PD cases [73, 83–86]. However, mitochondrial inclusions in the stellar ganglion cells of PD patients were not related to LBs formation or parkinsonism, raising the question as to whether they have a significance in the contest of PD. Decreased glucose consumption, likely reflecting a decrease in neuronal activity, has been reported in the nigrostriatal system of PD patients [87]. The augmented oxidative metabolism requirements, which have been detected in the PD brain by magnetic resonance studies, in conjunction with energy unbalance, were hypothesized to be indicative of mitochondrial dysfunction mechanisms that may be present in the brain of patients with PD even in the absence of overt clinical manifestations [88]. However, later studies failed to confirm deficiencies in the mitochondria ETS [89]. PD patients were found to show striatal oxidative stress directly relating to the progression of disease severity [90], suggesting that it may constitute a sign of synaptic deficits. Indeed, interruption of the activity-driven local ATP synthesis, an autoregulated mechanism that relies on proper synaptic functioning, can impair synaptic metabolism and induce the onset of functional deficits [91].

Yet, from these findings it can be concluded that although mitochondrial alterations have been reported in PD patients and disease models, it is not clear whether they represent a primary pathogenic mechanism. In particular, the critical interplay between mitochondrial dysfunction and oxidative stress, which has been widely reported in PD [92] and could constitute either a cause or a consequence of mitochondrial damage, hampers an effective comprehension of the abovementioned studies. Oxidative stress can constitute a bridge connecting mitochondrial dysfunction to the induction of *α*-synuclein misfolding, aggregation, and accumulation, but otherwise it may be also triggered by these latter events that in turn could induce mitochondrial alterations [67, 92].

Another relevant feature strictly related to mitochondrial dysfunction is intracellular calcium rise. MPTP and rotenone act by altering mitochondrial function with consequent calcium release from these organelles [67]. Interestingly, calcium rise and oxidative stress cooperatively promote *α*-synuclein aggregation [93–95]. On the other hand, *α*-synuclein can control mitochondrial calcium homeostasis by enhancing endoplasmic reticulum-mitochondria interactions [96] and its oligomerization exacerbates calcium dysregulation by increasing mitochondria permeability transition [97]. Therefore, could it be that mitochondrial deficits precede the onset of *α*-synuclein pathology in PD?

## 4. Evidences in Support of ***α***-Synuclein Synaptic Pathology Preceding Mitochondrial Dysfunction in PD

Both the loss of function and deposition of *α*-synuclein can significantly impact mitochondrial activity. Alpha-synuclein knockout mice have been found to present mitochondrial lipid abnormalities and electron chain impairment, suggesting that this protein is important for the control of mitochondrial function [98]. Interestingly, postmortem analysis of subjects diagnosed as premotor PD has revealed that the formation of LBs precedes the onset of mitochondrial damage, even though several brain areas affected by *α*-synuclein pathology show signs of oxidative stress and endoplasmic reticulum (ER) abnormalities [99]. LB-positive midbrain neurons in the PD brain present increased mitochondrial DNA damage but a direct causative link between respiratory chain dysfunction and protein aggregation has never been confirmed [100]. Accumulation of wild-type *α*-synuclein decreases mitochondrial complex I activity and increases the production of ROS in the mitochondria of human dopaminergic neurons and in the PD brain [101] as supported by the fact that the protein can promote mitochondrial deficits and oxidative stress [102]. In addition, *α*-synuclein can regulate mitochondrial fission, as its expression in mammalian cells, including neurons, both* in vitro* and* in vivo*, causes the fragmentation of mitochondria via direct interaction with their membranes. This fragmentation is eventually followed by a decline in respiration and neuronal death [103]. Alpha-synuclein overexpression in cells and C. Elegans reduces the fusion rate of mitochondria [104]. However, later studies showed that the protein is not present in mitochondria but localizes in a distinct portion of the ER, the mitochondria-associated ER membranes (MAM), and modulates mitochondrial morphology, a function that is impaired by pathogenic mutations in *α*-synuclein [105]. Despite *α*-synuclein levels hardly affect mitochondrial morphology in normal cell lines but may have some influence on that under certain environmental conditions [106], its accumulation can induce mitochondrial dysfunction in experimental* in vitro* models of PD [107, 108]. Overexpression of A53T mutant *α*-synuclein in cortical neurons causes marked deficits in mitochondrial motility, function, and dynamics that were found to be reversed by induction of *α*-synuclein autophagic clearance by rapamycin treatment [109]. In *α*-synuclein transgenic mice and in the brains of PD patients mitochondrial dysfunction has been found to be related to the reduction of TOM40, a specific component of mitochondria transport machinery. This coincides with increased mitochondrial DNA deletions and oxidative DNA damage as well as with decreased energy production and altered levels of complex I proteins [110]. Moreover, transgenic neuronal nonselective overexpression of *α*-synuclein in mice can induce mitochondrial dysfunction, preferentially within nigrostriatal dopaminergic neurons, long before the onset of striatal dopamine loss [111]. This latter finding suggests that other adjuvant pathological factors may render dopamine neurons more sensitive to *α*-synuclein deposition and may contribute to the onset of mitochondrial deficits within these cells. Indeed, *α*-synuclein knockout mice are less sensitive to mitochondrial toxin [112–114] thus confirming a key involvement of this protein in controlling sensitivity to mitochondrial deficits. Mitochondrial dysfunction associates with increased oxidative stress and *α*-synuclein accumulation in iPSC-derived neurons from Parkin-associated familial PD patients and postmortem brain tissue [115]. Furthermore, mutant A53T transgenic mice develop neuronal mitochondria degeneration with accumulation of *α*-synuclein-containing mitochondria and marked reduction of complex IV activity [116]. It is likely that whether *α*-synuclein accumulation starts at the synapse and consequently damages axonal transport in light of its loss of function, mitochondria neuronal trafficking may be significantly altered in the PD brain. On this line, neuronal-like cells with an inherent mitochondrial impairment derived from PD patients contain *α*-synuclein oligomers accumulation and a disorganized microtubule network [117]. Interestingly, the *α*-synuclein-associated mitochondrial-induced damage in these cells can be rescued by improving microtubule-mediated traffic [118], supporting that axonal damage links *α*-synuclein synaptic deposition to the onset of mitochondrial dysfunction.

Another important clue to take into consideration is the fact that *α*-synuclein has been found to interact with and modulate some members of the Rab GTPases protein family, such as Rab11 and Rab5 and Rab3a [119–122], which are important regulators in vesicular trafficking as well as endocytic and secretory pathways. Worth of note, among mitochondria quality control mechanisms such as mitophagy, AAA proteases, proteasomal degradation, and lysosomal-mediated mitochondrial vesicle cargos degradation [77], mitochondrial release from neurons is fundamental to ensure transcellular degradation of these organelles when they are damaged [123]. Therefore, impairment of Rab11 and Rab5 function induced by *α*-synuclein aggregation may lead and contribute to a block of the release of damaged mitochondria from neurons, thus resulting in their accumulation, a phenomenon that could in turn compromise cell homeostasis thus leading to cell degeneration. Interestingly, alterations in mitochondrial distribution with formation of small clusters have been reported in the substantia nigra pars compacta of a juvenile PD case [124]. Collectively, these lines of evidence support that *α*-synuclein pathology may precede the onset of mitochondrial dysfunction in PD. Nonetheless, studies demonstrating that mitochondrial complex I inhibitors can induce *α*-synuclein pathological aggregation in the brain and in peripheral nerve cells offer important points of reflection.

## 5. Evidences in Support of Mitochondrial Dysfunction Preceding ***α***-Synuclein Synaptic Pathology in PD

The strongest evidence supporting that mitochondrial damage precedes the onset of *α*-synuclein pathology derives from studies on MPTP and rotenone models. Indeed, repetitive exposure of rodents and monkeys to these toxins via oral, intraperitoneal, intragastric, or nasal administration results in the pathological accumulation of *α*-synuclein in central as well as peripheral neurons [65, 125–130].

Mice deficient in the NF-*κ*B transcription factor family member c-Rel, which is known to regulate the expression of mitochondrial uncoupling protein 4 (UCP4) [131], a proton transporter localized on the inner membrane, develop an age-dependent levodopa-responsive parkinsonism with nigral *α*-synuclein aggregation and neuron degeneration, as well as striatal synaptic dysfunction [132]. In addition, glucose deprivation, an insult that perturbs mitochondrial function and induces mitochondrial Ca^2+^ release, can trigger *α*-synuclein pathological accumulation in neuronal cells [9, 41]. Mitochondrial defects, similarly to *α*-synuclein deposition, can crucially affect synaptic function and neuronal resilience. Indeed, mitochondrial inhibitors have been found to alter corticostriatal synaptic functions and the modulatory effect exerted by neurotrophins [133]. Alterations of the mitochondria respiratory chain may induce oxidative stress that in turn leads to lipid peroxidation of cellular and vesicular membranes at synaptic sites, thus resulting in neurotransmitter release dysfunctions. This situation could constitute the ideal environment prompting *α*-synuclein conformational changes, accumulation, and aggregation. On this line, it has been demonstrated that synaptic dysfunction can trigger the accumulation of *α*-synuclein [134]. Also, alterations of mitochondrial fission or dynamics can reduce synaptic mitochondrial load and impair neuronal function by hindering the proper energy demand to ensure synaptic function. Indeed, multiple mitochondrial behaviors, especially those regulated by neuronal activity and synapse location, determine their distribution in the axon [135]. Factors regulating mitochondrial fission are also involved in the control of synaptic maturation [136] suggesting that mitochondrial alterations may impair the correct organization of the synaptic proteome, where *α*-synuclein constitutes one of the most abundant components. All these observations seem to support that mitochondrial dysfunction can affect synaptic environment and consequently result in *α*-synuclein accumulation at this site.

## 6. Conclusion

Despite the explosion of research describing the contribution of *α*-synuclein synaptic pathology and mitochondrial dysfunction to PD, the establishment of a precise chronological order for these events in the neurodegenerative processes underlying the onset and progression of this disorder is still impossible at present.

**Figure 1:**
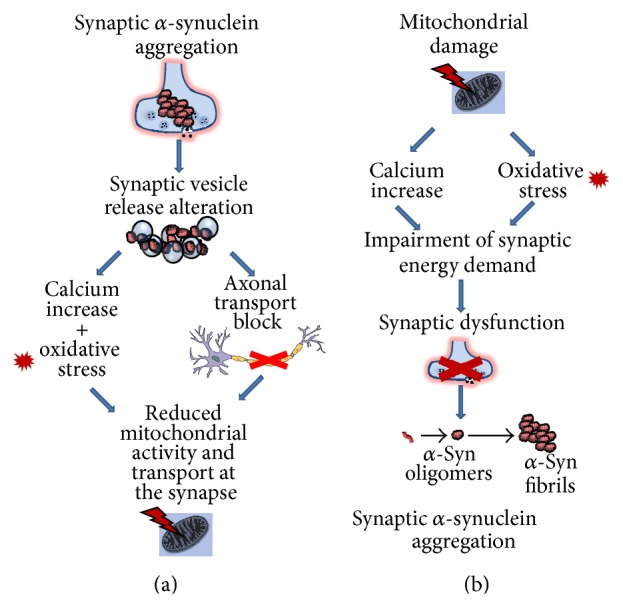
Hypothetical schematic representation of the two possible series of molecular events intervening either between synaptic *α*-synuclein deposition and the induction of mitochondrial dysfunction (a) or between mitochondrial functional deficits and accumulation of *α*-synuclein at the synapse (b). Please note that in both of the two situations, Ca^2+^ rise and production of oxidative stress mediators are pivotally involved in the interconnection between mitochondrial impairment and *α*-synuclein synaptic pathology.

However, as reviewed in this paper, although it is not clear “who's on first,” a series of clinical and experimental lines of evidence support the subsistence of a critical interconnection between these phenomena in the PD brain (Figure 1). Furthermore, they may very well trigger or foster other mechanisms of degeneration, thus reducing neuronal resilience. Yet, the available* in vivo* imaging techniques and experimental models, as well as the paucity of data from premotor PD, render it difficult to anticipate definitive conclusions on the relative contribution of these phenomena to disease. Nonetheless, it is clear that the pathways linking synaptic *α*-synuclein pathology and mitochondrial damage and vice versa must be investigated in deeper details to disclose novel important pathogenic players and therapeutic targets for PD.
